# Determinants of prenatal care use and HIV testing during pregnancy: a population-based, cross-sectional study of 7080 women of reproductive age in Mozambique

**DOI:** 10.1186/s12884-019-2540-z

**Published:** 2019-10-15

**Authors:** Sanni Yaya, Oladimeji Olarewaju, Kelechi Elizabeth Oladimeji, Ghose Bishwajit

**Affiliations:** 1 The George Institute for Global Health, 75 George Street, Oxford, OX1 2BQ United Kingdom; 2 Social Aspects of Public Health, Human Sciences Research Council, Cape Town, South Africa; 3 Department of Community Medicine, University of Jos, Jos, Nigeria; 4 Department of Global Health, School of Public Health, University of Namibia, Namibia, South Africa; 5 Department of Public Health, Faculty of Health Sciences, University of Fort Hare, Fort Hare, South Africa; 6 School of International Development and Global Studies, University of Ottawa, Ottawa, Ontario K1N 6N5 Canada

**Keywords:** Maternal health, Antenatal care, HIV test, Global health, Mozambique

## Abstract

**Background:**

In low-income countries with poor coverage of healthcare services such as Mozambique, antenatal care serves as a vital tool for providing life-saving and cost-effective services for pregnant mothers. Nonetheless, many countries in Africa, including Mozambique, are struggling to attain an optimum level of antenatal care (at least 4 visits) utilisation among pregnant women. In the present study, we aimed to assess the sociodemographic and economic factors associated with antenatal care use in Mozambique.

**Methods:**

Cross-sectional data from the latest round of Mozambique Demographic and Health Survey (2011) on women aged 15–49 years (*n* = 7080) were analysed. The outcome measures were early and adequate antenatal visit and HIV tests during the last pregnancy. Data were analysed using descriptive and multivariate regression methods. The predictor variables included various demographic (e.g. age, parity), empowerment (e.g. type of employment, household wealth status) and sociocultural factors (e.g. ethnicity, religion).

**Results:**

Of the 7080 women whose data was analyzed, 15.3 and 60.1% had early and adequate ANC visits respectively while 75.4% received HIV test during ANC visits. The odds of early ANC visits were higher [OR = 1.300, 95%CI = 1.062,1.592] among women in the rural areas compared with those in the urban areas. However, participants in rural areas had lower odds [OR = 0.788, 0.687,0.902] of receiving HIV tests during ANC visits. Women in the urban areas with secondary [OR = 1.296, 95%CI = 1.007,1.666] and higher [OR = 1.663, 95%CI = 1.052,2.628] education had higher odds of having early ANC visit. Those in the higher wealth quintiles also had significantly increased odds of using all three types of ANC indicators, particularly for rural women in the highest wealth quintile [OR = 4.776, 95%CI = 1.250,18.24]. Being within the higher wealth quintiles was found to significantly increase the odds of using all three types of ANC indicators, particularly women from rural areas with highest wealth quintile [OR = 4.776, 95%CI = 1.250,18.24].

**Conclusion:**

About two-fifth of the women in Mozambique are not using adequate antenatal care and about and a quarter do not take HIV tests during pregnancy. The sources of low and unequal use of these vital health services might be rooted in women’s socioeconomic status and cultural issues that require special policy and research attention.

## Background

As a part of overall human development process, countries in sub-Saharan Africa have been striving to reduce the high maternal and child mortality rates. Driven by the Millennium Development Goals (MDGs), many countries in the region have been able to make concrete improvements in this regard [1, 2]. Nonetheless, the sub-Saharan African countries continue to account for a staggering 66% of all pregnancy and childbirth related deaths in the world [3]. Maternal mortality is a complex indicator that can be triggered by a multitude of factors. However, it is widely acknowledged that the bulk of the health issues arising from pregnancy can actually be circumvented by taking precautionary measures provided through antenatal care techniques [4, 5]. Antenatal care (ANC) provides a unique window of opportunity to ensure a healthy pregnancy through ensuring healthy weight gain and proper nutrition (e.g. intake of iron and folic acid), taking necessary immunizations packages such as tetanus toxoid and antimalarial drugs, controlling gestational diabetes, hypertension, and many other pregnancy related medical conditions [4, 6, 7].

With the rising prevalence of childhood HIV, the impact of making ANC visits has become more prominent than ever as ANC service now includes HIV counselling and testing. Women who fail to attend ANC are less likely to be aware of the risks associated with mother-to-child transmission of HIV through pregnancy, childbirth, and breastfeeding. In the low-income settings, ANC visits allow women to check their HIV status which is an effective way to prevent mother-to-child transmission of HIV [8–10], the key contributor to childhood HIV in Africa [11, 12]. Exposure to ANC services not only increases the scope of taking the preventive measures and diagnosis of high-risk pregnancy and other pregnancy related conditions, but also the likelihood of choosing health facility delivery. Studies have demonstrated that majority of the maternal and child deaths occur during unassisted deliveries and in the neonatal period [13–15], which can be avoided if women are given the necessary consultations through the ANC package. This is particularly important for countries like Mozambique where a large proportion of the population live below the poverty line.

Nonetheless, a large proportion of the women across the developing world remain deprived of the ANC services [9]. The inequality in accessing and utilizing healthcare services is further attributed to various behavioral, cultural, economic, and sociodemographic factors at individual level and remoteness of health facility, inadequate infrastructure and skilled human resource for healthcare at community level [16–20]. Understanding a country’s specific predictors of healthcare utilisation is essential for making targeted approaches to combat pregnancy related deaths among women and newborns. However, there is no such study or report available for Mozambique, and thus the factors behind the low utilisation of ANC services are not known. Therefore, the present study was conducted to explore the factors associated with ANC service utilisation using nationally-representative data from Mozambique Demographic and Health Survey (DHS) conducted in 2011. This was a cross-sectional survey that provided information on a wide range of demographic and socioeconomic variables.

## Methodology

### Data source

Data for this study were collected from the sixth round of Mozambique Demographic and Health Survey. The survey was conducted in 2011 by the country’s national statistical institute (Instituto Nacional de Estatística) and ministry of health (MISAU). Techincal support and assistance was provided by United States Agency for International Development of the United America (USAID) with ICF International. Sample population included eligible men (15–54 years) and women (15–49 years) residing in households in urban and rural areas excluding institutions such as hospitals, hotels, dorms. Data collection was done through direct interviews using Tablet-type computer (Computer-Assisted Personal Interview) system and this process lasted from June 2011 to November 2011. Sampling was done using multistate cluster technique which involves stratifying the provinces into primary sampling units (PSUs), and then selection of households from the households form each PSUs. Of the 13,964 households initially selected, a total of 13,718 women were finally interviewed, resulting in a 99% response rate. These details are available from the final report of Mozambique 2011 DHS: Ministerio da Saude (MISAU), Instituto Nacional de Estatística (INE) e ICF International (ICFI). Moçambique Inquérito Demográfico e de Saúde 2011. Calverton, Maryland, USA: MISAU, INE e ICFI.

### Variables used in the analysis

Outcome variables.

The outcome variables of interest were: 1) timing of first antenatal care, 2) frequency of antenatal care, 3) HIV testing during ANC visit. All of these items were assessed based on the self-report of the participant for the latest childbirth occurring within the last five years from the survey. Timing of first antenatal care was classified as early (if within the first trimester) and late (if beyond the first trimester) [3]. Frequency of antenatal care visits was defined as adequate (at least four visits) and inadequate (less than four visits) as per World Health Organization recommendation. HIV testing during ANC visit was classified as Yes (had HIV tests) and No (had no HIV tests).

### Explanatory variables

Selection of explanatory variables was guided by Andersen’s behavioral model of health service utilisation which postulates that healthcare utilisation is a function of three major factors: 1) predisposing factors, 2) enabling factors and 3) need factors [21]. For this study, the data were secondary and hence the selection of the explanatory variables in line with the behavioral model was not possible. Based on the availability of variables in the dataset, the following was included in the analysis: Age (15–19, 20–24, 25–29, 30–34, 35–39, 40–44, 45–49); Residency (Urban, Rural); Education (No Education, Primary, Secondary, Higher); Husbands education (No Education, Incomplete Primary, Incomplete Secondary, Higher); Employment (Not Working, Professional/Technical/Managerial, Agricultural - Self Employed); Wealth quintile (Poorest, Poorer, Middle, Richer, Richest); Access to electronic media including TV and radio (No, Yes); Heard of FP on internet (No, Yes); Religion (Islam, Other); Ethnicity (Emakhuwa, Portugais, Xichangana, Cisena, Elomwe, Cindau, Xitswa, Other); Parity (1–5, > 5); Household head (Male, Female); Child wanted (Wanted Then, Wanted No More).

### Data management and analysis

The dataset was cleaned by applying the inclusion criteria: experience of at least 1 childbirth in the preceding 5 years and analysed with Stata version 14.. As the surveys used cluster sampling techniques, all analyses were adjusted for this by using the *svy* command [22]. This command uses the information on sampling weight, strata, and primary sampling unit provided with the datasets. Sample characteristics were described as percentages. Prevalence of timing and adequacy of antenatal care was presented as bar charts. Following that, binary logistic regression models were used to estimate odds ratio (with 95%CIs) of using these services. Results of three outcome variables were presented in separate tables, each divided into three subsamples: overall, urban and rural. Model fit statistics were run after the regression analyses using the variance inflation factor (VIF) command. No multi-collinearity was detected as VIF values were below 10 for all the models. All tests were two-tailed and were considered significant at alpha value of 5%.

### Ethical considerations

The analyses were done using publicly available data from demographic health surveys. Ethical procedures were the responsibility of the institutions that commissioned, funded, or managed the surveys. All DHS surveys are approved by ICF international as well as an Institutional Review Board (IRB) in respective country to ensure that the protocols are in compliance with the U.S. Department of Health and Human Services regulations for the protection of human subjects. an informed oral consent is obtained from each respondent and a parent or guardian provides consent for an adolescent under 18 years. The description of DHS consent process is available from https://www.dhsprogram.com/What-We-Do/Protecting-the-Privacy-of-DHS-Survey-Respondents.cfm.

## Results

### Summarized characteristics of the study participants

Table 1 shows that a greater proportion of the participants were aged 25–29 years (25.01%), rural residents (63.16%), had primary level education (51.5%), had no employment (53.59%), from households with richest wealth quintile (25.92%), had access to electronic media (71.69%), didn’t hear about family planning on internet (98.88%), followers of Islam (71.12%), of Xichangana ethnicity (31.87%), had 1–5 children (81.78%), from male-headed households (64.90%), and wanted the last child (79.86%).

**Table 1:** Summarized characteristics of the women aged 15–49 years (*N* = 7080)

Variable	Freq.	Percent
Age		
15–19	813	11.48
20–24	1771	25.01
25–29	1697	23.97
30–34	1319	18.63
35–39	935	13.21
40–44	394	5.56
45–49	151	2.13
Residency		
Urban	2608	36.84
Rural	4472	63.16
Education		
No Education	2139	30.21
Primary	3646	51.50
Secondary	1,22	17.23
Higher	75	1.06
Husband’s education		
No Education	1731	26.36
Primary	3272	49.82
Secondary	1422	21.65
Higher	143	2.18
Occupation		
Not Working	3794	53.59
Professional/Technical/Managerial	1269	17.92
Agricultural - Self Employed	2017	28.49
Wealth index		
Poorest	1067	15.07
Poorer	1194	16.86
Middle	1328	18.76
Richer	1656	23.39
Richest	1835	25.92
Electronic Media access		
No	2004	28.31
Yes	5076	71.69
Religion		
Islam	5035	71.12
Other	2045	28.88
Ethnicity		
Emakhuwa	1241	17.53
Portugais	607	8.57
Xichangana	1392	19.66
Cisena	676	9.55
Elomwe	266	3.76
Cindau	426	6.02
Xitswa	368	5.20
Other	2104	29.72
Parity		
1–5	5790	81.78
> 5	1290	18.22
Sex of household head		
Male	4595	64.90
Female	2485	35.00
Last child wanted		
Wanted Then	5654	79.86
Wanted No More	1426	20.14
Timing of ANC visit		
Early	5997	84.7
Late	1083	15.3
Frequency of ANC visit		
< 4	2825	39.9
4 or more	4255	60.1
HIV testing during ANC		
Yes	1742	24.6
No	5338	75.4

Table 1 also shaows that only 15.3% of the women made early contact, 60.1% had adequate number of ANC visits and 75.4% had HIV test during ANC visits.

Presented in Tables 2 and 3, are the sociodemographic predictors of early and adequate ANC visits respectively. As opposed to urban areas, in the rural areas women within aged 20–24 years significantly had lower odds of having early [OR = 0.634, 95%CI = 0.467,0.862] ANC visits (Table 2). The odds of early ANC visits were higher [OR = 1.300, 95%CI = 1.062,1.592] in the rural areas compared with urban (Table 2). Women in the urban areas with secondary [OR = 1.296, 95%CI = 1.007,1.666] and higher [OR = 1.663, 95%CI = 1.052,2.628] education had higher odds of having early ANC visit. Similar associations with educational status were observed for adequate ANC visit and taking HIV tests as well. No significant association was observed for adequate use of ANC (Table 3) among women aged 20-24 years, whereas a positive association was found between the age groups of 20–24 years and taking HIV test (Table 4).

**Table 2:** Predictors of making early ANC visits among women aged 15–49 years in Mozambique (*n* = 7080)

	Overall	Urban	Rural
Age (15–19)	1	1	1
20–24	0.711^*^ [0.547,0.924]	0.970 [0.572,1.645]	0.634^**^[0.467,0.862]
25–29	0.976 [0.755,1.261]	1.112 [0.662,1.867]	0.955 [0.707,1.291]
30–34	1.005 [0.769,1.313]	1.356 [0.801,2.293]	0.878 [0.638,1.207]
35–39	1.071 [0.796,1.441]	1.370 [0.782,2.397]	0.961 [0.669,1.379]
40–44	1.081 [0.766,1.526]	1.644 [0.852,3.170]	0.879 [0.581,1.330]
45–49	1.037 [0.658,1.634]	1.060 [0.424,2.646]	0.994 [0.583,1.696]
Residency (Urban)	1	NA	NA
Rural	1.300^*^ [1.062,1.592]		
Education (No Education)	1	1	1
Primary	1.181 [0.993,1.405]	0.929 [0.683,1.265]	1.332^**^ [1.077,1.648]
Secondary	1.256^*^ [1.054,1.497]	1.296^*^ [1.007,1.666]	1.158[0.900,1.491]
Higher	1.873^**^ [1.256,2.793]	1.663^*^ [1.052,2.628]	2.233 N [0.858,5.808]
Husband’s education (No Education)	1	1	1
Incomplete Primary	1.214[0.940,1.569]	1.291[0.869,1.917]	1.193[0.847,1.680]
Incomplete Secondary	1.070[0.907,1.261]	1.114[0.874,1.419]	1.044[0.829,1.316]
Higher	1.210[0.924,1.583]	1.438^*^[1.002,2.064]	0.977[0.638,1.498]
Employment (Not Working)	1	1	1
Professional/Technical/Managerial	1.036[0.884,1.214]	1.115[0.903,1.378]	0.930[0.725,1.194]
Agricultural - Self Employed	1.325^***^[1.141,1.538]	1.513^*^[1.019,2.247]	1.232^*^[1.039,1.461]
Wealth quintile (Poorest)	1	1	1
Poorer	1.052[0.891,1.243]	0.710[0.377,1.337]	1.096[0.921,1.306]
Middle	1.014[0.848,1.213]	0.914[0.550,1.518]	1.032[0.848,1.256]
Richer	1.048[0.829,1.325]	0.887[0.556,1.415]	0.932[0.652,1.332]
Richest	1.493^**^[1.143,1.951]	1.141[0.710,1.835]	4.776^*^[1.250,18.24]
Media access (No)	1	1	1
Yes	0.920[0.765,1.107]	1.157[0.692,1.934]	0.880[0.719,1.077]
Religion (Islam)	1	1	1
Other	1.166[0.719,1.888]	1.486[0.771,2.865]	0.757[0.357,1.603]
Ethnicity (Emakhuwa)	1	1	1
Portugais	0.661^***^[0.542,0.807]	0.857[0.614,1.195]	0.562^***^[0.436,0.724]
Xichangana	1.183[0.927,1.509]	1.126[0.796,1.594]	1.259[0.886,1.790]
Cisena	1.114[0.955,1.298]	1.057[0.787,1.420]	1.087[0.903,1.308]
Elomwe	1.105[0.874,1.397]	1.266[0.787,2.035]	1.002[0.762,1.319]
Cindau	1.168[0.951,1.435]	1.205[0.909,1.597]	1.185[0.861,1.632]
Xitswa	0.965[0.814,1.145]	1.051[0.704,1.567]	0.951[0.785,1.151]
Other	0.944[0.726,1.228]	1.073[0.527,2.185]	0.906[0.679,1.208]
Parity (1–5)	1	1	1
> 5	0.827^*^[0.691,0.989]	0.733[0.526,1.020]	0.901[0.723,1.123]
Household head’s sex (Male)	1	1	1
Female	0.984[0.835,1.160]	1.090[0.876,1.357]	0.867[0.669,1.122]
Child wanted (Wanted Then)	1	1	1
Wanted No More	0.705^***^[0.591,0.841]	0.847[0.641,1.120]	0.615^***^[0.488,0.775]
Pseudo *R*^2^	0.022	0.029	0.026

Exponentiated coefficients; 95% confidence intervals in brackets.

^*^ *p* < 0.05, ^**^ *p* < 0.01, ^***^ *p* < 0.001

**Table 3:** Predictors of making adequate ANC visits among women aged 15–49 years in Mozambique (n = 7080)

	Overall	Urban	Rural
Age (15–19)	1	1	1
20–24	0.953[0.786,1.155]	1.031[0.742,1.433]	0.916[0.722,1.162]
25–29	1.068[0.878,1.299]	1.171[0.836,1.640]	1.013[0.796,1.291]
30–34	1.001[0.812,1.234]	1.233[0.853,1.784]	0.895[0.692,1.157]
35–39	1.118[0.885,1.413]	1.500[0.993,2.267]	0.971[0.729,1.295]
40–44	1.098[0.818,1.472]	1.106[0.652,1.876]	1.065[0.747,1.519]
45–49	1.354[0.908,2.021]	2.157[0.878,5.297]	1.178[0.746,1.859]
Residency (Urban)	1		
Rural	1.021[0.884,1.181]		
Education (No Education)	1	1	1
Primary	1.149^*^[1.012,1.304]	1.282[0.956,1.720]	1.083[0.939,1.248]
Secondary	1.847^***^[1.476,2.313]	1.871^***^[1.301,2.691]	2.161^***^[1.509,3.093]
Higher	2.790^*^[1.141,6.827]	2.828^*^[1.105,7.241]	1.205[0.918,1.487]
Husband’s education (No Education)	1	1	1
Incomplete Primary	1.107[0.974,1.257]	1.002[0.762,1.317]	1.142[0.988,1.321]
Incomplete Secondary	1.219^*^[1.016,1.462]	1.186[0.876,1.605]	1.183[0.924,1.514]
Higher	1.970^*^[1.111,3.493]	2.012^*^[1.072,3.775]	1.317[0.137,12.63]
Employment (Not Working)	1	1	1
Professional/Technical/Managerial	1.159[0.989,1.357]	1.136[0.918,1.406]	1.253[0.981,1.601]
Agricultural - Self Employed	1.039[0.918,1.176]	1.023[0.767,1.364]	1.052[0.914,1.211]
Wealth quintile (Poorest)	1	1	1
Poorer	1.163[0.980,1.379]	1.573[0.890,2.783]	1.132[0.946,1.355]
Middle	1.404^***^[1.182,1.669]	1.623[1.000,2.635]	1.332^**^[1.105,1.606]
Richer	1.566^***^[1.302,1.885]	1.408[0.894,2.215]	1.496^***^[1.206,1.854]
Richest	2.100^***^[1.641,2.686]	1.955^**^[1.207,3.166]	2.871^***^[1.916,4.302]
Media access (No)	1	1	1
Yes	0.744^***^[0.660,0.840]	0.662^**^[0.504,0.870]	0.787^***^[0.687,0.903]
Religion (Islam)	1	1	1
Other	0.933[0.829,1.050]	1.105[0.888,1.377]	0.868[0.753,1.000]
Ethnicity (Emakhuwa)	1	1	1
Portugais	1.760^***^[1.326,2.335]	1.781^**^[1.219,2.601]	2.478^**^[1.327,4.626]
Xichangana	1.504^***^[1.226,1.845]	1.448^*^[1.033,2.029]	1.716^***^[1.308,2.251]
Cisena	1.376^**^[1.126,1.680]	1.875^**^[1.274,2.759]	1.215[0.959,1.541]
Elomwe	1.486^**^[1.123,1.967]	1.706[0.870,3.342]	1.419^*^[1.039,1.939]
Cindau	2.285^***^[1.773,2.946]	2.140^*^[1.135,4.032]	2.313^***^[1.746,3.064]
Xitswa	1.326^*^[1.014,1.732]	1.376[0.774,2.447]	1.312[0.967,1.780]
Other	1.580^***^[1.352,1.846]	1.760^***^[1.290,2.401]	1.544^***^[1.286,1.855]
Parity (1–5)	1	1	1
> 5	1.014[0.860,1.195]	0.864[0.623,1.198]	1.087[0.896,1.319]
Household head’s sex (Male)	1	1	1
Female	1.053[0.942,1.179]	0.900[0.741,1.092]	1.117[0.972,1.283]
Child wanted (Wanted Then)	1	1	1
Wanted No More	0.710^***^[0.617,0.818]	0.770^*^[0.626,0.948]	0.662^***^[0.544,0.806]
Pseudo *R*^2^	0.046	0.047	0.041

Exponentiated coefficients; 95% confidence intervals in brackets.

^*^ *p* < 0.05, ^**^ *p* < 0.01, ^***^ *p* < 0.001

**Table 4:** Predictors of HIV testing during ANC visits among women aged 15–49 years in Mozambique (*n* = 7080)

	Overall	Urban	Rural
Age (15–19)	1	1	1
20–24	1.232^*^[1.034,1.468]	1.129[0.687,1.855]	1.385^*^[1.056,1.816]
25–29	1.422^***^[1.191,1.697]	1.531[0.910,2.578]	1.687^***^[1.275,2.231]
30–34	1.376^***^[1.139,1.662]	1.806^*^[1.000,3.261]	1.521^**^[1.130,2.047]
35–39	1.283^*^[1.035,1.589]	1.232[0.651,2.331]	1.626^**^[1.163,2.273]
40–44	1.217[0.932,1.588]	1.016[0.444,2.325]	1.371[0.901,2.087]
45–49	1.149[0.799,1.652]	0.651[0.192,2.212]	1.302[0.755,2.248]
Residency (Urban)	1		
Rural	0.788^***^[0.687,0.902]		
Education (No Education)	1	1	1
Primary	1.373^***^[1.225,1.538]	2.011^***^[1.371,2.950]	1.338^***^[1.132,1.581]
Secondary	1.394^***^[1.176,1.652]	3.086^***^[1.733,5.493]	4.574^***^[2.604,8.035]
Higher	1.144[0.697,1.879]	6.053[0.641,57.18]	1.531[0.810,2.416]
Husband’s education (No Education)	1	1	1
Incomplete Primary	1.225^***^[1.088,1.378]	1.401[0.956,2.051]	1.306^**^[1.103,1.548]
Incomplete Secondary	1.453^***^[1.252,1.687]	2.549^***^[1.577,4.120]	1.350[0.992,1.836]
Higher	1.373^*^[1.006,1.874]	1.214[0.355,4.150]	1.126[0.854,1.832]
Employment (Not Working)	1	1	1
Professional/Technical/Managerial	0.861^*^[0.748,0.991]	1.784^**^[1.167,2.726]	0.702^*^[0.519,0.950]
Agricultural - Self Employed	0.744^***^[0.668,0.829]	0.857[0.580,1.265]	0.574^***^[0.487,0.675]
Wealth quintile (Poorest)	1	1	1
Poorer	1.062[0.933,1.209]	1.691[0.883,3.241]	0.999[0.817,1.222]
Middle	1.241^**^[1.082,1.422]	1.779^*^[1.011,3.131]	1.217[0.983,1.506]
Richer	1.429^***^[1.216,1.679]	1.955^*^[1.141,3.348]	1.533^**^[1.180,1.992]
Richest	1.959^***^[1.576,2.436]	2.534^**^[1.380,4.651]	3.376^***^[1.834,6.218]
Media access (No)	1	1	1
Yes	1.233^***^[1.102,1.381]	1.107[0.761,1.609]	1.111[0.947,1.303]
Religion (Islam)	1	1	1
Other	0.868^*^[0.767,0.983]	0.946[0.677,1.321]	0.821^*^[0.697,0.967]
Ethnicity (Emakhuwa)	1	1	1
Portugais	2.098^***^[1.737,2.533]	3.349^**^[1.602,7.000]	1.994[0.910,4.367]
Xichangana	2.072^***^[1.714,2.505]	3.006^***^[1.763,5.125]	1.689^**^[1.222,2.334]
Cisena	1.381^***^[1.206,1.581]	1.902^*^[1.104,3.275]	2.655^***^[2.030,3.473]
Elomwe	0.418^***^[0.315,0.555]	0.243^***^[0.111,0.533]	1.326^***^[1.153,1.526]
Cindau	1.415^**^[1.099,1.821]	1.281[0.847,1.938]	2.249^***^[1.831,2.762]
Xitswa	6.637^***^[4.489,9.813]	6.122^*^[1.393,26.90]	8.257^***^[5.379,12.67]
Other	3.168^***^[2.263,4.434]	3.143^*^[1.012,9.755]	3.778^***^[2.565,5.565]
Parity (1–5)	1	1	1
> 5	1.064[0.922,1.229]	1.013[0.622,1.650]	0.963[0.767,1.210]
Household head’s sex (Male)	1	1	1
Female	1.067[0.952,1.196]	0.856[0.621,1.179]	1.286^**^[1.085,1.524]
Child wanted (Wanted Then)	1	1	1
Wanted No More	0.643^*^[0.447,0.925]	0.599^**^[0.428,0.839]	1.144[0.894,1.464]
Pseudo *R*^2^	0.190	0.197	0.121

Exponentiated coefficients; 95% confidence intervals in brackets.

^*^ *p* < 0.05, ^**^ *p* < 0.01, ^***^ *p* < 0.001

Husband’s educational status also showed a positive association with women’s adequate ANC visits (Table 3) and taking HIV tests (Table 4). Women who had employment in agriculture were more likely to make early ANC in the both urban [OR = 1.513, 95%CI = 1.019,2.247] and rural [OR = 1.232, 1.039,1.461] areas.

For HIV tests, however, a significant negative association was found in urban women. Women in the higher wealth quintiles also have significantly increased odds of using all three types ANC indicators, particularly for rural women in the highest wealth quintile [OR = 4.776, 95%CI = 1.250,18.24]. Having access to electronic media showed an inverse relationship with adequate ANC use [OR = 0.744, 95%CI = 0.660,0.840], but positive relationship with taking HIV tests [OR = 1.233, 95%CI = 1.102,1.381]. Significant religion and ethnic variations were found for taking HIV testing services. Parity and household head’s sex didn’t show any noteworthy associations with the outcome variables.

A significantly negative association was found between child wanted-ness and the use of the ANC services. For early ANC visit, the negative association was true for women in the rural areas only [OR = 0.615, 95%CI = 0.488,0.775], and for taking HIV test the result was significant only in the urban [OR = 0.599, 95%CI = 0.428,0.839] areas. Whereas, having unwanted child showed a negative association with adequate ANC visits both in urban [OR = 0.770, 95%CI = 0.626,0.948] and rural [OR = 0.662, 95%CI = 0.544,0.806] areas. Women in rural areas had lower odds [OR = 0.788, 0.687,0.902] of receiving HIV tests during ANC visits (Table 4). As opposed to making adequate ANC vsits, having access to electronic media showed a positive relationship with taking HIV tests [OR = 1.233, 95%CI = 1.102,1.381].

## Discussion

Pregnancy represents a crucial phase of women’s reproductive life that requires a set of special care and services that can be accessed through antenatal care. This is especially true for women in low-income settings such as Mozambique where poor insurance coverage and high out-of-pocket expenditure can prevent the optimum use of these services. Research evidence is therefore necessary to understand the sociodemographic inequalities in the utilisation of ANC services in order to inform the ongoing programs working to meet the maternal health related targets such as Sustainable Development Goals. The current study provides an insight on the prevalence and predictors of the three crucial components of maternal healthcare including early and adequate antenatal care visits and use of HIV testing services among pregnant women in Mozambique. The findings indicate that only 15.3% of women made their first ANC visit within the first trimester, three-fifth had adequate ANC visits and three-quarter took HIV test during pregnancy. The prevalence of early ANC visits is lower than that of Ghana where 88% of the sample women received ANC care in 2014 [23]. In a multicountry study based on demographic and Health Survey data, Ghada et al. reported that the prevalence of having adequate ANC visits were Cameroon (62.7%), Senegal (51.2%), Uganda (48.5) [24]. Data on timing of the ANC visits is less commonly reported. In Ethiopia, a nationally-representative study found that 33.7% of the sample population received early ANC visits [3]. Regarding HIV tests during pregnancy, Ejigu et al. showed that in Ethiopia, more than 35% of the sample population were tested for HIV [25], which is quite low compared with 75.4% in the present study. This is understandable based on the fact that Ethiopia (2.4%) also has a far lower HIV rate compared with Mozambique (13.2% as of 2015) [26]. In fact, However, the prevalence of Mozambique women taking HIV tests during pregnancy is lower compared with Uganda (81.5%) [27] whose HIV rate is about half as much as in Mozambique [28].

In brief, the present study reports a low level of antenatal care use in Mozambique which requires special policy attention. Of particular concern is the prevalence of HIV testing, compared to the high incidence of HIV in the country the rate of HIV testing appears to be extremely low. Regarding the frequency and timing of ANC use, more than half of the women reported having at least 4 visits and making the first contact within the first trimester. Nonetheless, the number is still far from being universal as recommended by World Health Organisation. We found important sociodemographic inequalities for the use of these services, addressing whih can lead a better use of these life saving strategies. There are also several associations that deserve a special attention. For instance, women in the urban areas ANC were found to start ANC visit later than rural women, despite the fact that urban areas offer better access to healthcare. Although not deducible from the current findngs, this irregularity might be suugestive of urban poverty which is a growing issue across the continent. More research findings are necessary to understand this situation. Women who have outdoor employment were also less likely to get early ANC contact. In theory, working women are more empowered to access healthcare for themselves and for children. Therefore, this finding also remains to be interpreted from various perspectives such as healthcare benefits for pregnant women, granting leaves for checkup, and discrimination at healthcare centers. Collecting data on these factors are more complex and often challenging on a larger population, which is the likely reason why DHS studies do not collect data on these topics. New research models and techniques are required to meet these data limittaions.

Regarding the variables associated with the utilisation of the ANC services, several demographic (age), geographic (residency) and socioeconomic (education, wealth, ethnicity) factors were found be significantly associated with ANC service use. Women aged 20–24 years were found to have to lower odds of using timely and adequate ANC services compared with those in the lowest age group of 15–19 years. The odds of early ANC visits were higher in the rural areas compared with urban, however, rural women had lower odds of using HIV tests during ANC visits. The causes behind the regional differences were not possible to measure from the current analysis due to lack of contextual data. In general, it is believed that the prevalence of healthcare use is more prevalent among urban population compared with their rural counterparts due to better health awareness, financial capacity, transportation facilities. The causes behind the urban-rural disparity in the use of ANC services in Mozambique should be given priority in future studies.

In line with previous studies, women with better education status and coming from higher wealth quintile households had higher odds of making early ANC visits and taking HIV test [24, 27]. Women’s educational status is an important determinant of their social empowerment which itself is an important predictor of financial well-being and the capacity to access healthcare services. Education can promote healthcare seeking behaviour through its positive role on health awareness and improving self-efficacy [29]. Educated women are also more likely to be able to acquire information regarding the health risks associated with pregnancy which enable them to take preventive measures and reach out for professional helps such as counselling and medical tests. Similarly, being able to gather health information through modern electronic media such as radio, TV and internet can act as a strong enabling factor the healthcare use among women in the developing countries [30]. Strangely, the findings suggested no beneficial role of media access on making early and adequate ANC contact, although a positive association was found with taking HIV test. This supports the fact that women can gather knowledge on HIV transmission and risk factors and seek professional care during ANC as a means to prevent transmission of the virus to her child. Maternal health promotion programs in Mozambique should focus on developing strategies to improve the use of ANC services through leveraging mass media technology which is proving effective in many areas of public health interventions.

Women having employment in agriculture had a positive association with early ANC visits, but negative association with HIV test. Having an income source generally translates to better economic well-being and can act an enabling factor for utilisation of ANC services [27]. Maternal healthcare promotion programs should therefore pay special attention to non-working women who might remain deprived of the essential services owing to poor financial means. Another interesting finding was the ethnic disparity in the use of ANC services. Although the ethnicity is intrinsically a qualitative subject that requires qualitative investigation, our findings provide a glimpse of the relative inequality in the use of these services which can serve as the basis of further systematic investigation. Cultural factors influence health outcomes through various complex pathways, such as shaping people’s concept of health and illness and the perceived need for seeking medical care they may not be familiar with [31]. Sociocultural inequality in accessing healthcare is an increasing concern in populations as ethnically diverse as in Mozambique [32]. Therefore, it is recommended that healthcare policy making process be made more sensitive to the cultural factors that are generally ignored in the primary care settings. Lastly, we found a significantly negative association between child wanted-ness and use of ANC services. This is perhaps because of the lack of awareness and anxiety associated with unplanned pregnancy that prevent women from taking active steps in taking care of her and the pregnancy issues. Given the high rate of HIV in Mozambique, routine HIV tests during pregnancy should be regarded as a special priority to prevent the spread of childhood HIV.

This study made use of a nationally-representative survey to report the prevalence of using ANC services in Mozambique. In the current literature, data on healthcare use women during pregnancy is rare and insignificant. From this aspect, the present findings make an important contribution to the knowledge base and provide the basis for further research in this area. Nonetheless, this study has several limitations to report. First of all, the data were cross-sectional and hence no causality can be inferred from the associations. The data were secondary and authors have no influence over the selection and measurement of the variables. As the data were self-reported, the chances of recall and reporting bias cannot be ignored [33, 34].

## Conclusion

Findings of the present study highlights that although well above half of the women made at least four ANC visits and received HIV test during ANC, the proportions of women making early ANC contact was shockingly low (15%). There are important inequalities in using the services across various factors, most notable of which are education, wealth status, ethnicity, access to media wanted-ness of the child. Women who fail to make early ANC visits are more likely to have potential risk conditions unchecked which may give rise to serious complications at the later stage, therefore, policy makers and implementers should make it a priority to increase awareness on the importance of ANC and ensure contacts with ANC through clinic visits are enforced in the first trimester. Addressing the inequalities among the sociodemographic groups should be given special attention in maternal health promotion programs.

## Data Availability

Data for this study were sourced from Mozambique Demographic and Health surveys (DHS) and available here: https://dhsprogram.com/data/available-datasets.cfm

## Abbreviations

ANC: Antenatal care
DHS: Demographic Health Survey
IRB: Institutional Review Board
MDGS: Millennium Development Goals
USAID: United States Agency for International Development of the United America
