# Correction to: Determinants of prenatal care use and HIV testing during pregnancy: a population-based, cross-sectional study of 7080 women of reproductive age in Mozambique

**DOI:** 10.1186/s12884-019-2568-0

**Published:** 2019-11-21

**Authors:** Sanni Yaya, Olanrewaju Oladimeji, Kelechi Elizabeth Oladimeji, Ghose Bishwajit

**Affiliations:** 1 The George Institute for Global Health, 75 George Street, Oxford, OX1 2BQ UK; 2 Social Aspects of Public Health, Human Sciences Research Council, Cape Town, South Africa; 3 Department of Community Medicine, University of Jos, Jos, Nigeria; 4 Department of Global Health, School of Public Health, University of Namibia, Namibia, South Africa; 5 Department of Public Health, Faculty of Health Sciences, University of Fort Hare, Alice, Eastern Cape South Africa; 6 School of International Development and Global Studies, University of Ottawa, Ottawa, Ontario K1N 6N5 Canada

**Correction to: BMC Pregnancy and Childbirth (2019) 19:354.**

**https://doi.org/10.1186/s12884-019-2540-z**

Following publication of the original article [1], we have been notified that the name of the author two was spelled incorrectly as **Oladimeji Olarewaju**, when the correct spelling is **Oladimeji, Olanrewaju**.

Also a co-author affiliation was written as:

Department of Public Health, Faculty of Health Sciences, University of Fort Hare, Fort Hare, South Africa.

It should read as below:

**Department of Public Health, Faculty of Health Sciences, University of Fort Hare, Eastern Cape, South Africa.**

The original article has been corrected.
